# Effects of different lower-limb sensory stimulation strategies on postural regulation—A systematic review and meta-analysis

**DOI:** 10.1371/journal.pone.0174522

**Published:** 2017-03-29

**Authors:** Mei Teng Woo, Keith Davids, Jarmo Liukkonen, Dominic Orth, Jia Yi Chow, Timo Jaakkola

**Affiliations:** 1 Faculty of Sport and Health Sciences, University of Jyväskylä, Jyväskylä, Finland; 2 School of Sports, Health and Leisure, Republic Polytechnic, Singapore, Singapore; 3 Centre for Sports Engineering Research, Sheffield Hallam University, Sheffield, United Kingdom; 4 Faculty of Behavioral and Movement Sciences, Vrije Universiteit Amsterdam, Amsterdam, Netherland; 5 Physical Education and Sports Science, National Institute of Education, Nanyang Technological University, Singapore, Singapore; University of Florida, UNITED STATES

## Abstract

Systematic reviews of balance control have tended to only focus on the effects of single lower-limb stimulation strategies, and a current limitation is the lack of comparison between different relevant stimulation strategies. The aim of this systematic review and meta-analysis was to examine evidence of effects of different lower-limb sensory stimulation strategies on postural regulation and stability. Moderate- to high- pooled effect sizes (Unbiased (Hedges’ *g*) standardized mean differences (SMD) = 0.31–0.66) were observed with the addition of noise in a Stochastic Resonance Stimulation Strategy (SRSS), in three populations (i.e., healthy young adults, older adults, and individuals with lower-limb injuries), and under different task constraints (i.e., unipedal, bipedal, and eyes open). A Textured Material Stimulation Strategy (TMSS) enhanced postural control in the most challenging condition—eyes-closed on a stable surface (SMD = 0.61), and in older adults (SMD = 0.30). The Wearable Garments Stimulation Strategy (WGSS) showed no or adverse effects (SMD = -0.68–0.05) under all task constraints and in all populations, except in individuals with lower-limb injuries (SMD = 0.20). Results of our systematic review and meta-analysis revealed that future research could consider combining two or more stimulation strategies in intervention treatments for postural regulation and balance problems, depending on individual needs.

## Introduction

During human postural control, individuals constantly regulate movements, subconsciously, based on perceived information to achieve postural stability. In the past two decades, many studies have been devoted to investigating effects of lower-limb sensory stimulation strategies (e.g. tapes/sleeves/ braces/compression; application of Stochastic Resonance; textured insoles and footwear) for postural regulation and balance performance in different populations. Common to intervention strategies is the assumption that lower-limb stimulation increases somatosensory feedback. Specifically, it has been proposed that increasing attunement to plantar cutaneous afferent information sent to the Central Nervous System (CNS) [1,2] enhances balance/postural regulation by supporting faster detection of, and/or responses to, postural sway [3–7]. Studies have shown that cutaneous afference plays an important role in maintenance of balance [3,8–11]. Kavounoudias et al. [8] suggested that plantar cutaneous afference from the soles of the feet could potentially provide valuable feedback for balance regulation. They interpreted the sole of each foot as having a “dynamometric map”, where numerous sensors of the sole are able to spatially organise different pressures exerted against it, providing information to regulate balance and posture. Hence, plantar cutaneous afferents provide valuable feedback to the balance control system making it an important mechanism in supporting the functionality of the somatosensory system overall. Here we sought to examine evidence of the effects of different lower-limb sensory stimulation strategies on postural regulation and stability in order to contrast their relative effectiveness.

To the best of our knowledge, no published article has focused on the comparison of the effects of various lower-limb stimulation strategies such as tapes, braces, compression garments, textured insoles and footwear, and application of added random sub-threshold electrical or mechanical stimulation (noise) (exploiting a mechanism referred to as Stochastic Resonance [SR]). To date, systematic reviews have tended to only focus on a single stimulation strategy such as the role of textured materials [12], shoes and ankle appliances [3] and ankle taping and bracing [13]. Two of these studies quantified the review in a meta-analysis [12,13]. These studies have provided an overview of the effects of specific lower-limb stimulation strategies on balance control, but have been limited in not comparing the relevance of different stimulation strategies. A systematic review and meta-analysis of the entire body of work on lower-limb stimulation strategies will provide much-needed insights on the relevance of each stimulation strategy and its influence on postural regulation during static and dynamic balancing tasks. This information would inform clinicians, physiotherapists, practitioners, rehabilitation therapists and sport and exercise scientists about the relative effectiveness of each stimulation strategy.

The specific purposes of this review article are: i) to review systematically the effects of lower-limb stimulation strategies on sensory regulation of postural control and balance performance ii) to meta-analyze the effect of these lower-limb stimulation strategies on various populations (healthy young adults; older adults; individuals with lower-limb injuries) and under different task constraints (unipedal; bipedal; eyes open; eyes-closed).

### Previous evidence of efficacy of different lower-limb stimulation strategies: Wearable Garments (WG), Textured Materials (TM) and application of Stochastic Resonance (SR)

#### Wearable Garments (WG)

The main focus of early research using wearable garments (e.g., tapes, braces and compression garments) was related to injury and re-injury prevention [14–16]. Investigations centred on how disrupted proprioception could be enhanced by using wearable garments. For example, two studies investigated effects of strips and athletic tapes on joint movement position awareness [15,16] for injury prevention. Robbins et al. [15] suggested that the traction of the tape on the skin provided cutaneous sensory cues of plantar surface position to enhance anticipation of the foot position before contact with the floor. Similarly, effects of wearing compression garments (shorts and sleeves) on joint position sense [17] and postural regulation and balance during a single-leg landing task [14] were also tested. Kuster et al. [14] revealed that the compressive sleeve intervention reduced postural sway in the anteroposterior (AP) direction. It was argued that wearable garments such as tapes, compression garments, and braces could improve proprioception, specifically on improving judgment of ankle position and orientation of the plantar surface with respects to the leg [16, 18].

Furthermore, Kraemer et al. [17] suggested that it was possible that cutaneous receptors interacted with compression garment fabric, and that the signals may be even more important in fatigued musculature as evidenced by the test garments’ enhancement of performance in a fatigued jump task. They suggested that compression might interact with a biological cueing system to enhance performance. Following these promising findings, a number of research programmes have since examined the utility of different approaches for achieving improved performance outcomes, ostensibly via similar mechanisms. Such interventions have included: tapes [19–21], braces [22–24] and compression garments [25–27] in young, healthy adults and people with lower-limb injuries (e.g., Functional Ankle Instability (FAI)).

Michael [28] suggested that compression shorts improved the total time in unipedal standing balance in a more challenging condition involving visual occlusion. For young athletes with lower extremity injuries, studies have shown that compression via orthoses and sleeves improved uni- and bipedal balance performance [25,29–31] and proprioception [14, 20]. However, Papadapoulos et al. [24] suggested that ankle braces with 30kPa and 60kPa pressure were not able to alter the balance control strategy of the central nervous system (CNS). In an elderly sample, localized compression on the ankle was shown to improve joint position sense, but not a static balance performance [26]. Thus, mixed findings exist on the role of wearing compression garments, compared to control conditions of wearing non-compression garments on postural regulation.

#### Textured Materials (TM)

Textured materials, especially textured insoles, have also emerged as an important research area in somatosensory intervention studies. Texture insoles are deemed to be beneficial because they increase the sensitivity of plantar cutaneous afferent information sent to the CNS [1]. The systematic review by Hijmans et al. [3] suggested that insoles with tubing or vibrating elements might improve balance. However, no definitive conclusions have been drawn on effects of such insoles on balance in older people (> 60 years old) and patients with peripheral nervous system disorders. In a later meta-analysis carried out by Orth and colleagues, it was concluded that simple stimulation of cutaneous receptors via added texture can improve perceptual-motor system functionality in elderly individuals as well as in young, healthy adults. Several studies have suggested a positive relationship between balance/postural regulation and somatosensory feedback provided by use of textured insoles [4,5,7,32]. Qiu and colleagues [7] suggested that textured insole surfaces, both hard and soft, reduced postural sway during standing in older people. At the same time, Losa lglesias and colleagues [5] also reported that hard insole surfaces may be more effective compared to soft insole surfaces for reducing fall risk. Losa lglesias et al. [5] concluded that more rigid insoles promoted a more neutral alignment of the talocrural joint in a standing position, limiting the range of foot pronation, thereby, improving the ankle joint stability.

#### Stochastic Resonance (SR)

In addition to wearable garments and textured insoles, the use of sub-threshold electrical or mechanical stimulation (white noise) for stimulating soles of the feet, known as application of SR, has also received substantial research attention. Various studies introducing sub-sensory electrical or mechanical noise to enhance postural regulation were conducted between 2002 to 2014 (e.g., Collins et al. [33]). They examined effects of various intensities of electrical noise on balance control in FAI patients [34–36], older adults [37,38] young and healthy adults [39–42]. The majority of these studies found significant improvements in postural regulation during the performance of static and dynamic balancing tasks. These findings suggested that stimulation, through added noise, boosted detection of sensorimotor signals through SR, subsequently enhancing functioning of the postural regulation system. Sejdic & Lipsitz [43] stated that people who lost the resiliency and adaptive capacity due to aging and diseases, the postural regulation system can be restored by exploiting the phenomenon of SR.

From the motor system perspective, two studies highlighted the potential benefits of introducing SR in the motor systems of humans and cats [44,45]. Cordo et al. [44] showed how introduction of weak input signals (non-zero level of noise) through the tendon of a parent muscle enhanced the sensitivity of the muscle spindle receptors. This stimulation increased the muscle spindle sensory outputs to modulate performance in complex motor tasks (e.g., for balance). Martínez et al. [45] tested the effects of noise on the monosynaptic reflect pathway of the cat spinal cord. Their study showed that an application of SR could increase the sensitivity of motor neurons and that mechanical noise could be employed to improve feline motor task performance.

The majority of the reviewed studies showed promising findings on effects of individual lower-limb stimulation strategy on postural regulation systems. This quantified review seeks to provide new insights on this area of work with specific focus on the heterogeneity, similarities and differences between intervention strategies in enhancing postural regulation systems.

## Method

### Search strategy

The search and reporting format were conducted in accordance with the PRISMA statement (S1 PRISMA Checklist) [46]. Electronic databases (EBSCO, Science Direct, PubMed, Taylor and Francis, Google Scholar, and Scopus) were searched to identify publications concerning the effects of different lower- limb stimulation strategies (tapes/braces/sleeves, textured materials, and application of Stochastic Resonance) on postural regulation and stability. A detailed literature search identified articles published in English between 1995 and October 2016. An additional hand-conducted search of reference lists was undertaken to identify studies not captured in the electronic database searches. The following combination of two groups of keywords was used in searching relevant articles:

1. Lower-limb stimulation strategies: “Textured/Textured insoles or footwear”, “Compression/Compression garments and stocking”, “Tapes and braces”, “Application of Stochastic Resonance/ added noise.”
2. Task- related: “Postural Control” and “Balance.”

### Selection of studies

The first reviewer (lead author) performed the search of the electronic databases and screened the potentially relevant articles based on abstracts and titles at the initial screening. Then, the retrieved articles were evaluated separately by the first and fourth authors using the following inclusion and exclusion criteria for full review, with any disagreement resolved by consensus.

Inclusion Criteria:

1. No restrictions on study design.
2. Studies published in English between 1995 and October 2016.
3. Studies investigating the effects of behavioural measures of textured materials (insoles; stocking), wearable garments (compression garments; braces; tapes), and application of Stochastic Resonance during tasks involving postural stability and balance (static; dynamic) in non-fatigue conditions.
4. The primary outcome measures consisted of the center of pressure (CoP) related measurements, the center of mass (CoM), distance reach, balance time, and gait variables.
5. The primary outcomes included in the meta-analysis were the center of pressure (CoP) related measurements such as CoP sway and standard deviations (SD) in medial-lateral (ML), and anterior-posterior (AP) directions; path length; recurrence quantification analysis (RQA) measurements.
6. In the meta-analysis, studies were required to report means and standard deviations of outcome measures interacting with lower-limb stimulation strategies.

Exclusion Criteria:

1. Studies that use cumbersome and expensive equipment in investigating vibration effects on postural ability, which is more complex and costly for end-users, compared to the simpler addition of electrical stimulation, textured, and wearable materials. Furthermore, vibration effects usually produce stimulation above the consciously perceived threshold level, which would negate the role of Stochastic Resonance in enhancing proprioception and haptic perception at a sub-threshold level.
2. Studies where outcome variables are not compatible for comparison, (means and standard deviations) in the meta-analysis.
3. Studies in stroke, diseases resulting in neuropathy (e.g. Multiple Sclerosis and Parkinson Disease) and cerebral palsy populations, with some damage to the brain, impairing physical mobility and postural control mechanisms.

### Lower-limb stimulation strategies

In this context of study, three main groups of the lower-limb stimulation strategies were based on the characteristics—wearable garments, textured materials, and an application of stochastic resonance. Compression garments (sleeves; socks), braces and tapes were grouped as Wearable Garments (WGSS). Textured insoles and footwear were grouped as Textured Materials (TMSS). Last, implementation of white noise and electrical stimulation were grouped as an application of Stochastic Resonance (SRSS).

### Assessment of methodological quality

The methodological quality of the included studies was assessed by two reviewers (lead author and the fourth author) using the Cochrane Collaborations tool for evaluating the risk of bias [47]. The domains of assessment were sequence generation, allocation concealment, blinding of participants, personnel and outcome assessors, incomplete outcome data, selective outcome reporting and other sources of bias. Summary outcomes of all studies for each domain were categorised as “low risk of bias”, “high risk of bias” and “unclear risk of bias”. Any discrepancies were resolved through consensus between the two reviewers.

### Data extraction and management

For the systematic review, the extracted data included: sample size, participant characteristics, tasks, equipment used, balance-related measurements and main outcomes of the study. Only studies that reported the outcome measures of interest (CoP related measurements) were included in the statistical analysis. The CoP related data was used due to its commonality in postural control and regulation research [48]. Furthermore, Ruhe et al. [49] also suggested that 81.3% of the research studies demonstrated acceptable reliability levels of using CoP measures and it could be used as a reliable tool for investigating general postural stability and balance performance under specific conditions.

### Analysis and meta-analytic techniques

The primary analysis was to compare the effectiveness of the three different lower-limb stimulation strategies—Wearable garments, Textured materials, and application of Stochastic Resonance. Unbiased (Hedges’ *g*) standardized mean differences (SMD) and 95% confidence intervals (CI) were calculated for continuous outcomes [50]. In studies that investigated more than two treatments (e.g., compression socks and normal socks), the calculated effect size of the multiple treatments was combined and treated as a single effect size for each continuous outcome [51]. Five studies from WGSS [23,24,27,28,52], four studies from TMSS [7,53–55], and three studies from SRSS [33,39,56] had more than two treatments in the experimental design. All the effect sizes of the outcomes were then combined as a single effect size for each study. In the event of multiple stages of task design (pre- and post-conditioning procedures, for example), the most appropriate stage (pre- or post-treatment) was chosen, based upon its relevance to the objective of this analysis.

For dependent group designs, effect size estimates for lower-limb stimulation strategies for the control group in each study were standardized using the control group standard deviation value [12]. However, the pooled standard deviation was used as the denominator in independent group designs [51]. The value of rho estimates from Orth et al. [12] was used to compute the unbiased variance estimates. Subsequently, standard error (SE) of each study was calculated based on the computed unbiased variance estimates [51].

Calculated synthesized (by average) estimated effect size and standard error (SE) values were imported into Review Manager (RevMan computer program, version 5.3.5 Copenhagen: The Nordic Cochrane Centre, The Cochrane Collaboration, 2014) for the calculation of pooled effect size, p-value, z- value, Tau^2^, heterogeneity (I^2^). The following settings were used: data type—generic inverse variance; statistical method—inverse variance; analysis model—random effects; effect measure—standardized mean difference (SMD); study and total confidence interval– 95%. An effect size of 0.1 is considered small, an effect size of 0.3 is medium, and an effect size of 0.5, large [51].

Consequently, pooled effect size, 95% confidence interval, P-value and heterogeneity (I^2^) were calculated per subgroup, per study. An alpha level of .05 (two-tailed) was used to test whether the average effect size was significantly different from zero in each subgroup.

#### Subgroup analysis

The subgroup analysis was used to derive pooled estimates of the three subgroups (WGSS; TMSS; SRSS) for the differences observed on postural control performances. Three main areas identified for subgroup comparison were: i) static balance tasks—single-leg standing (SLS) and double-limbs standing (DLS); ii) populations—young and healthy adults, older adults, lower-limb injuries individuals (e.g. Ankle sprained; Anterior Cruciate Ligament reconstruction; Knee Osteoarthritis); and iii) vision availability—eyes open and -closed. There is always some debate over the measurements of postural sway to express system stability. In the meta-analysis of this review, decreases in CoP measurements imply the increased ability of a postural-regulation system to maintain balance. Hence, a positive effect size indicated a functional role of stimulation strategies, while a negative effect size inferred that there was a positive functional capacity of the control group in regulating posture.

## Results

### Study selection and characteristics

**Fig 1:**
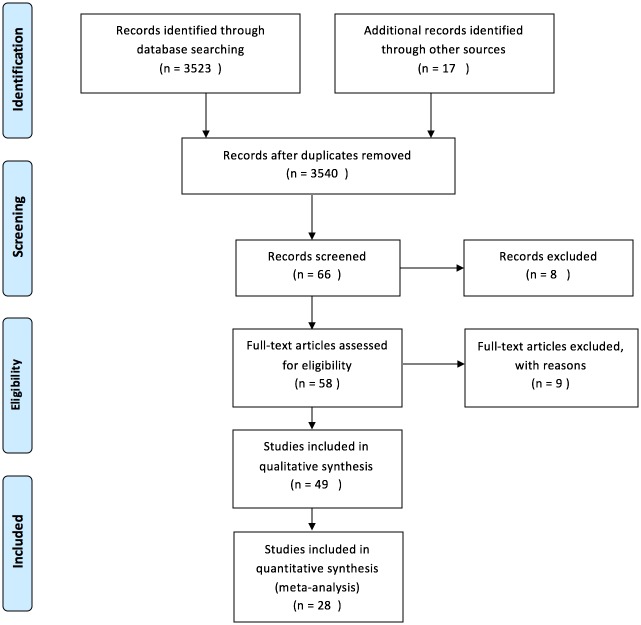
Summary of the search strategy and selection process based on included and excluded studies.

Fig 1 outlines the process of the literature review search. A total of 58 out of 3540 studies were identified, based on the title and abstract review from the electronic database and hand searches. Nine studies were excluded as the intervention treatments, or the measure outcomes did not match the inclusion criteria after the initial assessment.

Of the 49 studies (S1 Table), 18 examined the effects of the wearable garments stimulation strategy (WGSS), 19 investigated the effects of textured materials stimulation strategy (TMSS), and 12 studied the application of Stochastic Resonance stimulation strategy (SRSS). Thirty studies used a repeated measures design, with each participant performing in all the conditions (control and stimulation treatments). Seventeen studies used a mixed-model design, where they were two or more independent groups performing all the treatment conditions (S1 Table). Six studies used a pre, post-test protocol, where the intervention periods ranged from 5-minutes to 12-weeks, to measure the effects of lower-limb stimulation strategies [37,57–61]. One study, by Qu [62], was unique in that it did not specify a control condition to compare with the other textured insoles treatments.

A summary of the characteristics of all included studies was tabulated according to the three different lower-limb stimulation strategies and presented, based on participant characteristics (Table 1) and the assessment tasks (Table 2). In Table 1, the majority of studies (n = 28) involved young and healthy adults as research participants to examine the effect of the lower-limb strategies on static and dynamic balancing tasks. The second main population for the wearable garments’ research included participants with some form of lower-limb injuries (n = 7). The textured material (n = 5) and application of Stochastic Resonance (n = 4) research studies focused on a middle/old age population.

**Table 1:** Characteristic of included studies based on populations.

Lower limbs' strategies	Study	Young Healthy Adults	Middle-aged and older adults	Neuropathy patients	Athlete / Elite	Lower limbs' Injuries or medical condition	Remarks
Wearable Garments	Birmingham et al. (2001)					√	
	Broglio et al. (2009)	√					
	Cavanaugh et al. (2016)	√					
	Genthon et al. (2010)					√	
	Gribble et al. (2010)					√	
	Hadadi et al (2011)	√				√	
	Hadadi et al. (2014)	√				√	
	Hijmans et al. (2009)	√	√				
	Kunzler et al. (2013)	√					
	Kuster et al. (1999)					√	
	Michael et al. (2014)				√		
	Ozer et al. (2009)	√					
	Palm et al. (2012)					√	
	Papadopoulos et al. (2007)	√					
	Sperlich et al. (2013)				√		
	Vuillerme & Pinsault (2007)	√					
	Wheat et al. (2014)	√					
	Woo et al. (2014)		√				
Textured Materials	Aruin & Kaneka. (2013)	√					
	Collings et al. (2015)	√					
	Corbin et al. (2007)	√					
	Hatton et al. (2009)	√					
	Hatton et al. (2011)		√				
	Hatton et al. (2012)		√				
	Jenkins et al. (2009)		√	√ (PD)			
	Kelleher et al. (2010)	√		√ (MS)			
	Ma et al. (2016)	√					
	Maki et al. (1999)	√	√				
	Menz et al. (2006)	√	√	√			
	Palluel et al. (2008)	√	√				
	Palluel et al. (2009)	√	√				
	Perry et al. (2008)		√				
	Qiu et al. (2012)	√	√				
	Qiu et al. (2013)		√	√ (PD)			
	Qu (2015)		√				
	Stern & Gottschall (2012)	√					
	Wilson et al. (2008)		√ (middle-aged)				Mean age: 51.1±5.8
Stochastic Resonance	Amiridis et al. (2005)		√				
	Collins et al. (2012)		√			√ (Knee Osteoarthritis)	
	Dickstein et al. (2005)	√					
	Gravelle et al. (2002)		√				
	Kimura & Kouzaki (2013)	√					
	Magalhaes & Kohn (2012)	√					
	Magalhaes & Kohn (2014)	√					
	Ross (2007)					√	
	Ross & Arnold (2012)					√	
	Ross & Guskiewicz (2006)	√				√	
	Ross et al. (2007)					√	
	Ross et al. (2013)	√					

**Table 2:** Characteristic of included studies based on tasks constraints.

Lower limbs' strategies	Study	Static			Dynamic		Gait		
		SLS	DLS	Tandem Stance	Unstable/ moving platform (SLS); SEBT	Single leg land_Jump/hop	Stable	Uneven	Hill
Wearable Garments	Birmingham et al. (2001)	√***; √ ^#^^##^				√			
	Broglio et al. (2009)	√*	√*	√*					
	Cavanaugh et al. (2016)				√ (SEBT)	√			
	Genthon et al. (2010)		√**						
	Gribble et al. (2010)					√			
	Hadadi et al (2011)	√*							
	Hadadi et al (2014)				√ (SEBT)				
	Hijmans et al. (2009)		√***						
	Kunzler et al. (2013)		√**						
	Kuster et al. (1999)					√			
	Michael et al. (2014)	√***							
	Ozer et al. (2009)	√							
	Palm et al. (2012)				√				
	Papadopoulos et al. (2007)	√***							
	Sperlich et al. (2013)				√				
	Vuillerme & Pinsault (2007)		√**						
	Wheat et al. (2014)	√***							
	Woo et al. (2014)		√***; √ ^#^^##^						
Textured Materials	Aruin & Kaneka. (2013)		√*		√		√		
	Collings et al. (2015)						√		
	Corbin et al. (2007)	√***	√***						
	Hatton et al. (2009)		√*						
	Hatton et al. (2011)		√***						
	Hatton et al. (2012)		√***				√		
	Jenkins et al. (2009)						√		
	Kelleher et al. (2010)						√		
	Ma et al. (2016)						√		
	Maki et al. (1999)				√				
	Menz et al. (2006)		√***						
	Palluel et al. (2008)		√*				√		
	Palluel et al. (2009)		√*						
	Perry et al. (2008)							√	
	Qiu et al. (2012)		√***; √ ^#^^##^						
	Qiu et al. (2013)		√***; √ ^#^^##^						
	Qu (2015)		√***						
	Stern & Gottschall (2012)						√		√
	Wilson et al. (2008)		√***				√		
Stochastic Resonance	Amiridis et al. (2005)	√*	√*	√*					
	Collins et al. (2012)	√*							
	Dickstein et al. (2005)		√*						
	Gravelle et al. (2002)	√*							
	Kimura & Kouzaki (2013)		√**						
	Magalhaes & Kohn (2012)		√*						
	Magalhaes & Kohn (2014)		√**						
	Ross (2007)	√*							
	Ross & Arnold (2012)					√			
	Ross & Guskiewics. (2006)					√			
	Ross et al. (2007)	√*							
	Ross et al. (2013)	√*	√*						

* Vision: eyes open;

**Vision: eyes-closed;

***Vision: eyes open and closed;

^#^ Surface: stable;

^##^ Surface: stable and foam

From Table 2, the double-limb standing (DLS) balance tasks were the most commonly used tests in all studies (n = 24). Surprisingly, the single-leg standing (SLS) balance task has not been considered as the main assessment task in TMSS research. However, the TMSS grouping included the most number of studies in gait related tasks. Whereas, the wearable garments stimulation strategy (WGSS) grouping favored both static and dynamic balance tasks, specifically SLS. On the other hand, a majority of the studies in WGSS and SRSS examined only one task, while the TMSS grouping tended to include two or more tasks in a single study.

### Risk of bias

**Fig 2:**
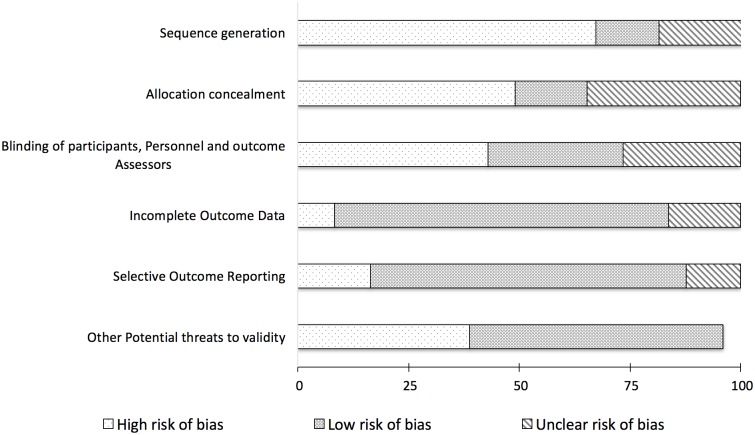
Risk of bias summary.

**Fig 3:**
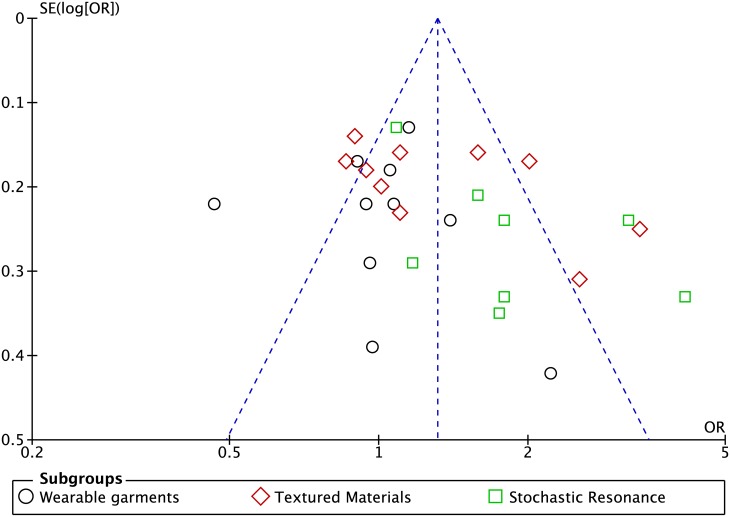
Funnel plot of all studies included in the meta-analysis.

Most studies in WGSS and TMSS groupings were at high risk of bias for selection bias, performance, and detection categories (Fig 2). Fig 3 depicts a funnel plot based on the 28 studies included in the meta-analysis on the effects of lower-limb stimulation strategies on postural control. The asymmetrical shape of this plot suggests possible reporting bias or low methodological quality of some studies [63].

Overall, about 67% of the studies (n = 33) met the criteria for high risk of bias in the sequence generation category. Of the 33 studies, SRSS had the least number of studies (n = 5; 15.2%) followed by TMSS (n = 11; 33.3%) and WGSS (n = 17; 51.5%). In the WGSS group, all 17 studies (100%) included a non-random approach for selection of participants. There were 24 studies meeting criteria for high risk of bias in the allocation concealment category. Of the 24 studies, 13 from WGSS (54.2%), 8 from TMSS (33.3%) and 3 from SRSS (12.5%).

With regards to the blinding categories, a high risk of bias was found in both the WGSS and TMSS groups– 13 (61.9%) and 6 (28.6%) out of 21 studies, respectively. However, it is important to acknowledge the challenge faced when attempting to blind participants and researchers to intervention materials that are perceivable, especially in WGSS and TMSS groups.

In contrast, most studies (~75.5%) were at as low risk of bias in the participant attrition and reporting categories, with no missing outcome data, with all the relevant dependent variables apparently reported. However, two studies each from WGSS [26,30] and TMSS [59,64] groupings were at high risk of bias for incomplete outcome data. The reasons for this included: imbalanced numbers of participants across the groups [26,59], or because participants were excluded due to an additional injury [30], or reduced total sample sizes due to data corruption [64].

Eight studies were rated at a high risk of reporting bias because they failed to report the key outcomes or provided incomplete information on dependent variables [1,7,20,28,34,59,65,66]. The reasons for this were due to incomplete outcomes reported [7,28,65], and failure to include results for key outcomes [1,20,34,59,65].

### Performance outcomes

Overall, a total of 30 studies (WGSS = 9; TMSS = 11; SRSS = 10) reported positive effects of applying lower-limb stimulation strategies on static balance tasks, dynamic balance tasks and gait (S1 Table). Five studies (WGSS = 1; TMSS = 3; SRSS = 1) concluded the findings with a neutral statement [61,67–70]. A study from WGSS reported mixed results, where they only found the beneficial effects in under specific constraints, including an eyes-closed condition [28]; and under a stability task performed by participants on an injured leg [22,23,25].

#### Wearable Garments Stimulation Strategy (WGSS)

Of the 7 studies in this grouping, six found that braces, tapes and compression sleeves improved postural regulation especially in participants with lower-limb injuries [22,23,25,30,71]. There was only one study by Gribble et al. [65] reporting that an ankle brace did not affect the balance performance in a population with a lower-limb injury.

For the young and healthy adults, Kunzler et al. [72] and Vuillerme and Pinsault [21] indicated that taping treatment improved postural regulation in a non-fatigue condition, as shown in the decrease of CoP velocity and CoP surface area. In contrast, eight studies did not find it beneficial by applying the tapes or braces on the limbs of young and healthy adults [19,20,23,24,26,52,67,71].

There were two studies that reported no significant differences in postural regulation with compression bandages and stockings for older adults [26,27]. Two studies examined the effects of compression garments on postural regulation in athletes [28,66]. Sperlich et al. [66] concluded that no significant improvement in postural regulation with the compression garments in the eyes-closed condition, whereas, Michael et al. [28] found otherwise.

#### Textured Materials Stimulation Strategy (TMSS)

A total of 10 studies examined TMSS effects on postural regulation in static and dynamic balance tasks in young and healthy adults. Five studies reported beneficial effects during performance in DLS balance tasks [1,57,58,73,74], four studies found no significant differences in postural regulation during double-limb standing [7,53] and in gait [69,75], and two suggested that the evidence was insufficient to arrive at a concrete conclusion for dynamic balance control (Gait) [68,70].

For middle-aged and older adults, 63.6% (7 out of 11) of the studies indicated that textured insoles had a positive influence on static balance control [7,54,57,58], dynamic balance control [74] and postural regulation in gait [59,64]. Three studies found no significant improvement or detrimental effects of using textured insoles on postural regulation during the double-limb standing tasks [55,60,62]. There was only one study showing no improvement in gait with the use of facilitatory ribbed insoles [76].

There was a unique study that examined the effects of using Velcro, an affordable and innovative approach to providing tactical stimulation, on three groups of participants—young and healthy adults, older adults, and diabetic peripheral neuropathy patients [56]. The Velcro was used to rub gently against the skin on the side of the ankle, calf and knee, instead of stimulating the soles of the feet. Findings revealed that the Velcro stimulus significantly reduced postural sway in all three groups of participants.

#### Application of Stochastic Resonance Stimulation Strategy (SRSS)

Ten out of twelve studies (83.3%) concluded that application of SR had a significant effect on postural regulation in young and healthy adults [39–42,77], older participants [37,38], and participants with lower-limb injuries [34–36]. Positive effects were observed in static balance tasks [34,35,37–42,77] and a dynamic balance task (Single-leg landing task) [36].

Only one study did not find a significant improvement in performance on a single-leg standing task with sub-threshold electrical stimulation in older adults with minimal-to-moderate knee osteoarthritis [33]. On the other hand, Ross & Guskiewicz [61] postulated that application of SR stimulation might improve dynamic postural stability more quickly than coordination training for lower-limb injuries (Functional ankle instability) participants.

### Meta-analysis

Among the included studies, 28 studies reported CoP measurement outcomes for the static balance tasks and were included in meta-analysis. The subgroups are wearable garments (n = 10), textured materials (n = 10) and application of SR (n = 8) as lower-limb stimulation strategies. For the static balance tasks, CoP measurements such as time-to-stabilization, path length, the postural sway velocity and distance in both Anterior-posterior (AP) and medial-lateral (ML) directions under the different vision (e.g., eyes open and closed) and surface (stable & foam) conditions were included in the analysis. For the sample populations (young and healthy adults, elderly individuals, and with lower-limb injuries), the CoP measurements from SLS and DLS under the stable surface condition with eyes open and -closed were included in the analysis. For the vision conditions, CoP measurements of all populations, which performed both SLS and DLS under the stable surface, were included in the analysis. Both SLS and DLS comprise popular experimental paradigms used by researchers. The reasons for their popularity could be due to the complex sensory information perceptual mechanisms used during upright stance. It could also due to the association between DLS and the risk of falling, especially in elderly population [78,79]. Single-leg standing was used to increase the difficulty of the standing task and also for a comparative examination of balance performance between the injured and non-injured legs.

#### Tasks: Single-Leg Standing (SLS) & Double-Limbs Standing (DLS)

**Fig 4:**
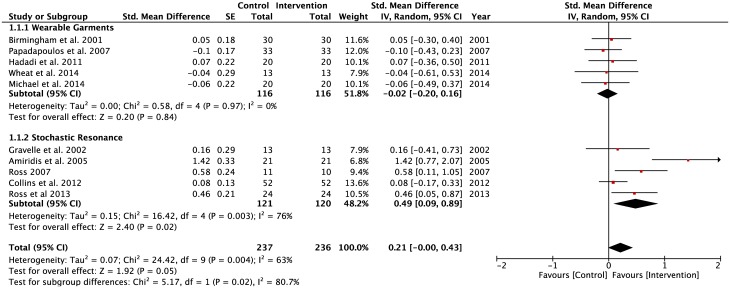
Forest plots for single-leg standing balance task. Task: Single-Leg Stand; Vision: Eyes open and closed; Surface: Stable and foam; Population: Healthy young; Older adults; Lower-limbs’ injuries.

Ten studies (WGSS = 5; SRSS = 5) reported CoP measurements of postural regulation during performance of the SLS task (Fig 4). There was a significant difference between the two subgroups, WGSS and SRSS (p = 0.02), where a higher pooled effect size was observed in SRSS group (SMD = 0.49, z = 2.40, p = 0.02). However, there was a high level of heterogeneity observed in the SRSS (Tau^2^ = 0.15; df = 4; I^2^ = 76%) group compared to the homogeneity observed in the WGSS group (Tau^2^ = 0.00; df = 4; I^2^ = 0%). This finding indicated that 76% of variability between effect sizes of SRSS studies displayed a systematic influence of one or more variables. The average pooled effect size for the WGSS was found to be 0.20, which corresponds to a small effect size. In comparison, the SRSS had a moderate effect size (SMD = 0.41). The overall application of both WGSS and SRSS during SLS had beneficial effects on postural regulation during performance of a SLS task on stable surfaces with eyes open and closed condition (z = 1.92, p = 0.05).

**Fig 5:**
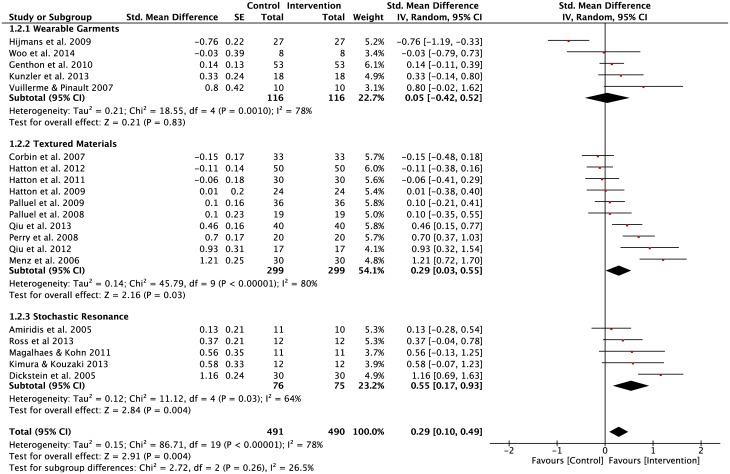
Forest plots for double-limbs standing balance task. Task: Double-limbs standing; Vision: Eyes open and closed; Surface: Stable and foam; Population: Healthy young; Older adults; Lower-limbs’ injuries.

For the DLS balancing task, a total of 20 studies (WGSS = 5; TMSS = 10; SRSS = 5) were included for analysis (Fig 5). The mean effect sizes of TMSS (SMD = 0.29) and SRSS (SMD = 0.55) showed low to moderate effects of implementation of lower-limb stimulation strategies on performance in the DLS balance task. In contrast, the WGSS (SMD = 0.05) barely showed any effects on postural regulation measurements during the DLS balance task. All three groups revealed significant levels of heterogeneity, suggesting that different population samples, vision and surface conditions, or experimental design-related factors influenced levels of variability across effect sizes. The findings showed beneficial effects when applying all three lower-limb stimulation strategies during performance in the DLS task on stable surfaces (z = 2.91, p = 0.004).

In summary, the SRSS is more effective intervention strategy compared to TMSS and WGSS under the SLS and DLS conditions.

#### Populations: Young and healthy, elderly individuals and lower-limb injuries individuals

**Fig 6:**
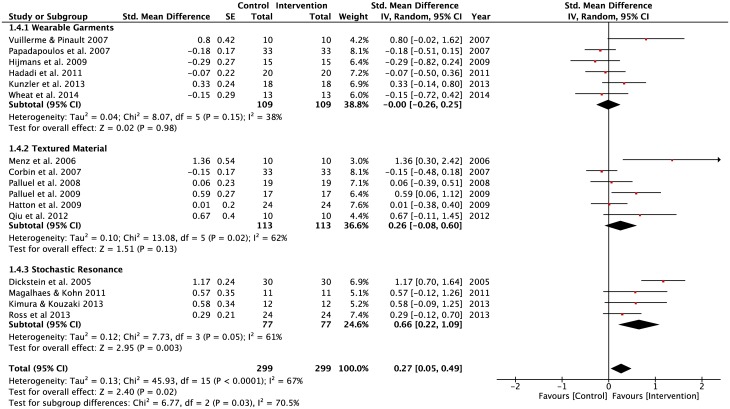
Forest plots for the young and healthy population. Population: Young and healthy; Vision: Eyes open and closed; Surface: Stable; Task: Single-leg and double-limbs standing tasks.

A total of 16 studies (WGSS = 6; TMSS = 6; SRSS = 4) that involved young and healthy adults, were included in the analysis (Fig 6). Of the three lower-limb stimulation strategy groupings, only the SRSS demonstrated a moderate positive effect (SMD = 0.66, p = 0.003) on postural regulation performance during static balance tasks—SLS and DLS on a stable surface with eyes open and closed. A forest plot showed that there was no effect demonstrated by WGSS (SMD = 0) and a low effect was observed in TMSS (SMD = 0.26). Both TMSS and SRSS showed moderate levels of heterogeneity: 62% & 67% respectively. There was an overall positive effect of applying SRSS observed in young adults on postural regulation during performance of static balance tasks on a stable surface with eyes open and closed (z = 2.95, p = 0.003).

**Fig 7:**
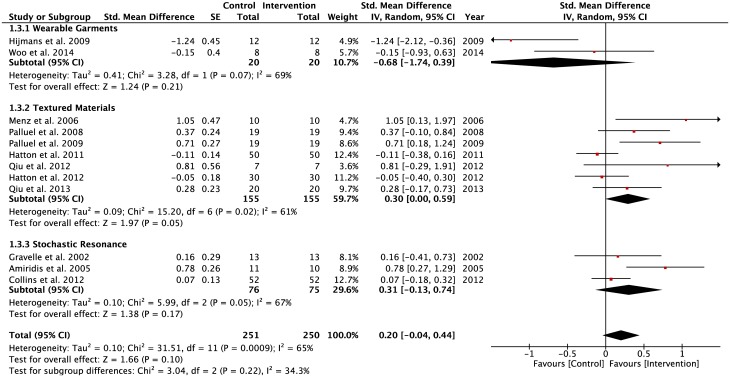
Forest plots for the older adults population. Population: Older adults; Vision: Eyes open and closed; Surface: Stable; Task: Single-leg and double-limbs standing tasks.

For the elderly individuals, a forest plot (Fig 7) showed that there was a moderate negative effect of wearing compression garments (SMD = -0.68). The pooled effect size for TMSS and SRSS appeared low (SMD = 0.30 and 0.31 respectively). There was evidence of moderate heterogeneity for WGSS (Tau^2^ = 0.41; df = 1; I^2^ = 69%), TMSS (Tau^2^ = 0.09; df = 6; I^2^ = 61%) and SRSS (Tau^2^ = 0.10; df = 2; I^2^ = 67%).

**Fig 8:**
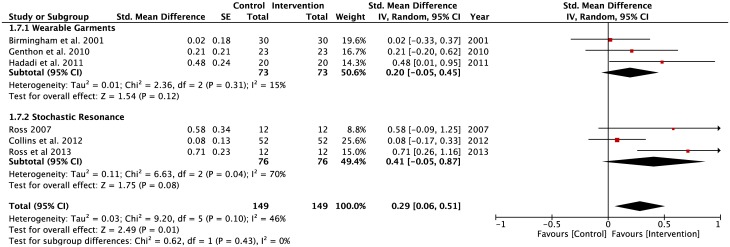
Forest plots for the lower-limbs’ injuries population. Population: Lower-limbs’ injuries; Vision: Eyes open and closed; Surface: Stable; Task: Single-leg and double-limbs standing tasks.

There were no differences across the lower-limb stimulation interventions on postural regulation during performance of static balance tasks on a stable surface with eyes open and eyes-closed (Fig 8). The pooled effect size values were low and moderate in WGSS (SMD = 0.20, z = 1.54, p = 0.12) and SRSS (SMD = 0.41, z = 1.75, p = 0.08) sub-groups, respectively.

In summary, the SRSS showed beneficial effects on postural regulation in all three populations. In contrast, the WGSS induced adverse effects in an elderly population.

#### Visions: Eyes open and—closed conditions

**Fig 9:**
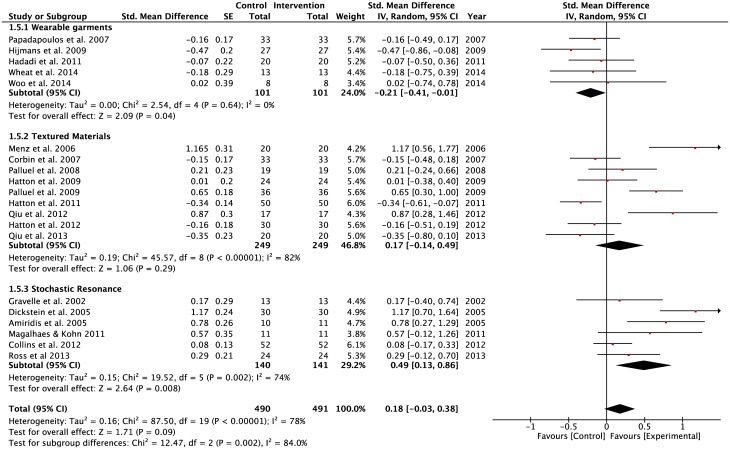
Forest plots for eyes open condition. Vision: Eyes open; Surface: Stable; Task: Single-leg and double-limbs standing tasks; Population: Healthy young; Older adults; Lower-limbs’ injuries.

A significant negative pooled effect size observed in the WGSS sub-group (SMD = -0.21, z = 2.09, p = 0.04) during static balancing tasks performed by participants on a stable surface under the eyes open condition (Fig 9). In contrast, the SRSS subgroup had a significant positive pooled effect size (SMD = 0.49, z = 1.71, p = 0.008) under the same task constraints. There were significant levels of heterogeneity in both the TMSS (Tau^2^ = 0.19; df = 8; I^2^ = 82%) and SRSS (Tau^2^ = 0.15; df = 5; I^2^ = 74%) subgroups. Conversely, homogeneity was observed in the pooled effect size of the WGSS subgroup.

**Fig 10:**
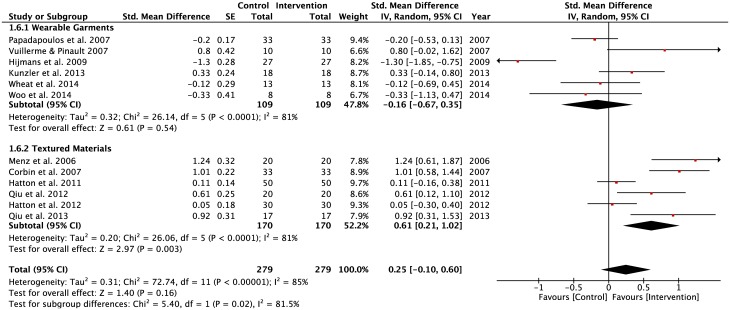
Forest plots for eyes- closed condition. Vision: Eyes- closed; Surface: Stable; Task: Single-leg and double-limbs standing tasks; Population: Healthy young; Older adults; Lower-limbs’ injuries.

A negative pooled effect size was observed in the WGSS sub-group (SMD = -0.16, z = 0.61, p = 0.54) for the static balance tasks performed by participants on a stable surface under the eyes closed condition (Fig 10). In contrast, the TMSS subgroup had a significant positive pooled effect size (SMD = 0.61, z = 2.97, p = 0.003) under the same task constraints. Of the 6 studies in the WGSS subgroup, only 2 studies demonstrated positive effects of interventions (Kunzler et al. [72]; Vuillerme & Pinsault [21]). There were significant heterogeneity levels in both the WGSS (Tau^2^ = 0.32; df = 5; I^2^ = 81%) and TMSS (Tau^2^ = 0.20; df = 5; I^2^ = 81%) subgroups.

In summary, WGSS did not have any beneficial effects for performance in the static standing task on a stable surface under both vision conditions. In contrast, SRSS & TMSS showed beneficial effects under the same conditions.

## Discussion

The primary aim of this quantitative review was to investigate effectiveness of different lower- limb sensory stimulation strategies on postural regulation through systematic review and meta-analysis. Of additional interest was the comparison of effects with respect to three major subgroupings in the extant literature—WGSS, TMSS and SRSS in various populations (young and healthy; older adults; individuals with lower- limb injuries) and under different task and informational constraints (Unipedal; Bipedal; Eyes open; Eyes -closed). To the best of our knowledge, this is the first systematic review and meta-analysis comparing the effectiveness of various lower-limb stimulation strategies on postural regulation performance in different sub -populations.

Qualitative and quantitative analyses of SRSS studies showed significant positive effects of applying a SR strategy in young and healthy adults during static balance tasks (SLS & DLS) and in a vision condition. In contrast, a quantitative analysis of WGSS effectiveness showed no, or adverse, effects in older adults, young and healthy populations, during performance of static balance tasks (SLS & DLS), with eyes open and closed conditions. However, the qualitative analysis demonstrated some beneficial effects of using WGSS such as braces, tapes and compression sleeves. The quantitative and qualitative analyses suggested that TMSS studies revealed small and moderate positive effects of wearing textured materials on postural regulation during static and dynamic balance tasks.

### Effects of Wearable Garment lower-limb Stimulation Strategies (WGSS)

Qualitative data suggested that wearable garments provide additional somatosensory information, inducing cutaneous pressure, probably by enhancing stimulation of plantar cutaneous receptors in the lower limbs [14,17,21,22,25,72], except in the elderly population. The quantitative outcomes of the review showed positive effects of wearable garments on postural control under static task constraints, revealing that enhanced performance was more prominent in people with lower-limb injuries [14,22,23,25,30,71] compared to a young, healthy people [19,20,23,24,26,52,71], and older adults [26,27]. These observations suggest that the disruptive effects of injury or illness on mechanoreceptors in an injured limb can be ameliorated by increasing stimulation of the cutaneous receptors through taping, braces and wearing compression garments. However, this was not the case for young, healthy adults, with few observed positive effects in quantitative analyses. This could be that “healthy postural control systems” may not benefit from the additional stimulation provided by wearable garments.

Only two studies, by Kunzler et al. [72] and Vuillerme and Pinsault [21] demonstrated a moderate to high positive effect size in applying the medical and athletic adhesive tapes in young and healthy adults during performance in the DLS balance tasks. In these studies, the tapes were placed on the anterior aspect of the ankle and along the Achilles tendon, unlike in other studies, where the entire ankles were wrapped/covered up by the braces, tapes or socks. Hence, it is possible that the pressure applied by the tapes in these two particular locations provided a more effective stimulant to the ankle joint receptors as well as the skin receptors in the lower limb. Further studies are needed to investigate location-specific effects of worn garments on motor task performance, to evaluate this possibility. It is plausible that the athletic taping could be used in sports that require high balance capacity such as ice dancing, Alpine skiing and gymnastics.

Studies by Hijmans et al. [26] and Woo et al. [27] are the only two studies to have examined the effect of compression garments (bandage and socks) on postural regulation in older adults. Hijmans et al. [26] found a main effect of compression, with balance significantly disturbed in the elderly aged 85.4 ± 4.6 years old. However, Woo et al. [27] concluded that both compression and commercial socks displayed similar functions in the regularization of anterior-posterior (AP) and medial-lateral (ML) motions, as seen under barefoot conditions for the elderly participants aged 70.9 ± 5.9 years old. The age groups of the participants for these two studies were different, which might explain the slight differences in the outcomes. Furthermore, the small sample size used in Woo et al. could have affected the reported outcomes. Nevertheless, these two studies left us with the question on whether wearing compression garments (e.g. socks and bandages) provide beneficial or deteriorating effects in influencing postural regulation in daily living activities in elderly populations remains unknown. Hence, more research is needed to investigate the effects of wearable garments on individuals of various ages in elderly populations.

### Effects of Textured Materials lower-limb Stimulation Strategies (TMSS)

The qualitative review showed that double-limb standing balance measurements were most commonly used in studies of textured materials. Additionally, participants were almost always older adults. The quantitative review showed that textured insoles enhanced postural regulation performance in challenging conditions—during upright balance with eyes closed on a stable surface (SMD = 0.61), in older adults (SMD = 0.30).

The motivations behind these studies are rooted in ideas of deterioration of sensory system performance and the concept of sensory systems re-weighting their contributions to action regulation. Changes in cutaneous sensitivity and receptor morphology have been observed as people age, and have been associated with a reduction in neural activity, which increases the sensory and vibration threshold needed for accurate perception [27,59,79]. Studies included in this review reflect the role of textured insoles in enhancing the sensorimotor signals to provide plantar cutaneous afferent information in older adults during balance and postural regulation. These effects indirectly supported by evidence of textured insoles in improving postural regulation under DLS task constraints [7,54,57–59,64,74].

From the perspective of sensory re-weighting, individuals primarily rely on the somatosensory inputs when visual information is unavailable [80]. Presner-Domjan et al. [80] proposed that mechanical stimulations such as textured insoles could activate the plantar cutaneous receptors to compensate the loss of visual information. The idea of sensory re-weighting is supported by the quantitative analysis in this review, where high pooled effect size values (SMD = 0.61) were observed in the review of studies investigating effects of textured insoles on postural regulation during static balance tasks with eyes closed. This finding highlights an area requiring more research in the future, especially with regards to individuals with visual impairments and elderly people with eye problems (e.g. Cataract & Glaucoma). Furthermore, it might imply that such wearable technology might enhance somatosensory feedback to athletes during performance (e.g in gymnastics, skiing, football) and reduce the dependence on visual information during the competitive performance.

Of the 4 studies [7,55,56,59] that reported high positive effect sizes in favour of wearing added textured materials, the study by Menz et al. [56] is the only one that used velcro to stimulate the mechanoreceptors and skin receptors at either the ankle, calf, or knee. These findings prompt the question whether other sensory receptors in the lower limbs, besides the cutaneous receptors from the soles of the feet, could also be stimulated to enhance postural regulation system function. Furthermore, future research might consider altering task constraints, including difficulty levels of balance tasks (e.g., unstable with eyes open and -closed conditions), as noted in the studies by Qiu and colleagues’ in 2012 [7] and 2013 [55].

It is also worth noting that the TMSS was the only lower-limb stimulation strategy not used to investigate the effects of textured materials on participants with lower-limb injuries. Previous studies have revealed beneficial effects seen in DLS task performance in other populations. It is plausible that people with lower-limb injuries could benefit from using the textured materials to help their balance and postural control. The findings of this systematic review and meta-analysis suggest that textured materials could be potentially used as a medium to ameliorate negative effects on the postural control system due to aging.

### Effects of applying Stochastic Resonance as a lower-limb Stimulation Strategy (SRSS)

The qualitative analysis showed that 83.3% of the studies reported that imperceptible electrical stimulation (white noise, 0.01mA– 0.05mA) enhanced balance control, being associated with reduced postural sway during performance in static balance tasks. This stimulation strategy showed moderate- to high-pooled effect sizes in most of the populations and categories studied—young and older adults, healthy individuals, static balance tasks, and with vision available.

The current quantitative review focused on research studies with a common site of stimulation along the shank, and between the ankle and knee joints. Most of the electrodes were placed on the muscles, ligaments and bone (lateral and medial side of femoral condyles). It is postulated that muscle spindles and mechanoreceptors along the shank were sensitive to the stimulation by SR. Stimulation of these sites yielded beneficial effects on postural regulation in older adults, and in patients with lower limb injuries, as well as healthy young adults. A key difference in studies, noted from the review, concerns variations in the sites of stimulation, focusing on particular receptor(s). The majority of the studies in the WGSS grouping applied wearable garments at the ankle joint (targeting joint receptors). The soles of the feet (cutaneous receptors) were the most common stimulation sites used in TMSS studies. For the SRSS research, the site of stimulation was typically between the knee and the shank (targeting muscle spindles; mechanoreceptors). With the moderate to high effect sizes reported in the SRSS studies, investigators might wish to consider other sites of stimulation in future WGSS and TMSS studies.

The direct application of weak input signals (non-zero level of noise) enhanced the detection of sensorimotor signals, which were beneficial to motor task performance (e.g., balance) [44,45]. Our review article revealed that none of the previous studies measured the effects of SRSS on athletic performance and using athletes as the study sample (Table 1). Hence, this highlights another research areas for future studies to consider implementing this lower-limb stimulation strategy in sports contexts that require good balancing in performers, such as, competitive cycling, kicking a ball, skiing, snow and skateboarding and surfing.

Of three studies [39,40,42], the study by Kimura and Kouzaki [40] was the only one that reported the necessary information for effect size calculation in an eyes-closed condition. The moderate effect size (SMD = 0.58) prompts speculation that SRSS might also be effective under a condition where reliance on somatosensory system information is intensified by removal of available visual information. This suggests the possibility of using Stochastic Resonance to enhance somatosensory system signals and reduce the reliance on visual information for basic postural control. Future research could consider including an eyes-closed condition to measure the effects of applying SR on somatosensory system function and its effects on visually impaired populations.

### Limitations and future research

A limitation of this review is that some outcomes included summary values extracted from graphs when values were not reported, reflecting estimations of the treatment effects. This review solely focused on research that had undergone rigorous, external peer review in international, scientific journals. The varied intervention periods in the 4 studies included in the meta-analysis might have had some impacts on the overall effects of the respective lower-limb stimulation strategies.

The findings of this review suggest three possible areas for future research. First, this review suggests that WGSS, TMSS and SRSS applications could be extended to the study of older adults, people with lower-limb injuries, as well as with elite and developing athletes, since many sports require attunement to information from the lower limbs for successful performance. Second, task performance difficulty levels need to be included in future analyses of experimental effects, such as the use of unstable surfaces in an eyes-closed condition and walking on uneven surfaces to measure the effectiveness of the lower-limb stimulation strategies. Third, at a later stage, there is a need to study effects of integrating two lower-limb stimulation strategies such as wearing textured and compression materials (or analysing the relative influence of different compression levels [clinical and non-clinical levels]) on the somatosensory system function in sports and clinical settings, particularly for developing athletes and in people with significant sensory function disorders.

## Conclusion

A review of current evidence in published literature indicates that an SRSS has produced the most effective results in postural regulation, compared to implementing interventions with wearable garments and textured materials. An SRSS achieved moderate to high effect size in all the populations and task constraints studied—healthy young and older adults, single-leg and double-limbs standing balance tasks and eyes open condition. Regardless of these differences, the costs of the organising specific interventions also need to be considered. The review revealed that the WGSS was effective in studies of patients with lower-limb injuries, and TMSS was found to be beneficial in young and healthy population in a double-leg standing task. Future research can consider to investigate the effects of textured materials in populations with lower limb injuries during performance on a single- leg standing task. The usage of SRSS and WGSS could be extended out to neuropathy patients and elderly population respectively. Furthermore, researchers could use at least two of the currently used stimulation strategies in combination as an intervention treatment for people with significant sensory function disorders, or for the enhancement of skill and expertise in elite and developing athletes. The combination of two stimulation strategies might yield better results by enhancing the sensorimotor signals to the nervous system to support performance.

## Supporting information

S1 PRISMA Checklist PRISMA 2009 checklist. (DOC)

S1 Table Characteristic of included studies. (DOCX)
