# Supplementary material for: Effects of different lower-limb sensory stimulation strategies on postural regulation—A systematic review and meta-analysis
Source: PLoS One. 2017 Mar 29;12(3):e0174522. doi: 10.1371/journal.pone.0174522 (PMC5371369; doi:10.1371/journal.pone.0174522)
Supplement: S1 Table — (DOCX) [file pone.0174522.s002.docx]

| **Table 1: Characteristic of included studies** | | | | | |
| --- | --- | --- | --- | --- | --- |
| **Lower limbs' stimulation strategies** | **Study** | **Study Design** | **Equipment** | **Comparison** | **Outcome** |
| Wearable garments | Birmingham et al. (2001) | Repeated measures | Force platform (AMTI model OR6-5) | Brace vs no brace treatments | A significant main effect for the brace condition. Knee brace improved the control of single-limb standing balance ^+ve^ |
|  | Broglio et al. (2009) | Repeated measures | Balance Error Scoring System (BESS) NeuroCom Sensory Organisation Test (SOT) | Barefoot vs prophylactic ankle taping vs laced bracing treatments | Ankle taping and laced bracing had negative influence on BESS postural control performance before and after a 20-min walk. ^-ve^ |
|  | Cavanaugh et al. (2016) | Repeated measures | Y Balance Test; 50cm drop jump landing (dominant leg) | Kinesiology tape vs Knee Compression stocking vs control  Fatigue vs non-fatigue conditions | There was no statistically different among the treatment conditions - kinesiology tapes, compression sleeves and control on balance tasks performance. ~ |
|  | Genthon et al. (2010) | Mixed-design | Force platform (PF02, Equi+, Aix les Bains) | Barefoot vs compression stocking (15-20mmhg) vs orthosis (internal elastic taping) treatments | Ankle orthosis reduced the postural instability of ankle sprain patients. The mean resultant CoP trajectories amplitude was reduced during compression stocking. ^+ve^ |
|  | Gribble et al. (2010) | Repeated measures | Force platform (Bertec) | Brace vs no brace treatments | There was no significant influence of the selected ankle brace on the Resultant vector of time-to-stabilisation (RVTTS) in single-limb landing balance task. ^-ve^ |
|  | Hadadi et al (2011) | Mixed-design | Force platform (Bertec) | Healthy adult vs Functional ankle instability (FAI) patients No-brace vs soft vs semi-rigid treatments Injured vs non-injured limb | Interaction of foot and brace significant in the FAI group not the healthy group. ^+ve^ A significant effect of brace was found only for the injured limb. ^+ve^ Decreased in postural sway in FAI group especially in soft brace condition. ^+ve^ Postural sway increased from no-brace condition to soft then to semi-rigid orthoses.^+ve^ |
|  | Hadadi et al (2014) | Mixed-design | Star Excursion Balance Test (SEBT) | Healthy vs Functional ankle instability (FAI)  No-brace vs soft vs semi-rigid treatments Injured vs non-injured limb | Ankle orthoses improved reach distance in FAI patients in various reach directions. ^+ve^ |
|  | Hijmans et al. (2009) | Mixed-design | Foot pressure plate (Rsscan Footscan) Modified Slope box Pressure monitoring device | No compression bandage vs compression bandage class II (23-32 mmhg) treatments | Balance deteriorated in older participants with the application of compression. ^-ve^ Joint position sense improved significantly in older participants as a result of the application of compression. ^+ve^ |
| Wearable garments | Kunzler et al. (2013) | Repeated measures | 3-D force plate (OR6 2000, Advanced Mechanical Technology) | No-tape vs tape treatment Fatigue vs non-fatigues conditions | Tape improved postural control in the non-fatigue condition as shown in the decrease of CoP velocity and CoP amplitude. ^+ve^ |
|  | Kuster et al. (1999) | Repeated measures | Force platform (Kistler) | Non-compression sleeve vs elastic compression sleeve treatment | A 10% increase of peak ground contact forces when wearing compression sleeves. ^+ve^ A significant reduction was observed in the path length of the CoP (indicating improved steadiness). ^+ve^ A compression sleeve seems capable of compensating for the loss of proprioception and muscle coordination after ACL rupture or reconstruction by compressing the skin, muscles and tendons. ^+ve^ |
|  | Michael et al. (2014) | Repeated measures | Force platform (Kistler) Motion Analysis System (Vicon) | Conventional shorts vs loose-fitted compression garment vs well-fitted compression garments treatments | By wearing the well-fitted compression garment significantly improved balance time and significantly decreased postural sway variability compared with conventional shorts in the **eyes closed condition.** ^+ve^ Compression garments had no effect on static balance when **vision was present**. ^-ve^ |
|  | Ozer et al. (2009) | Repeated measures | Chronometer Functional squat system machine | Barefoot vs preventive brace (Aircast) vs prophylactic tape treatments | There was no significant difference among the treatments for balance test. ^-ve^ |
|  | Palm et al. (2012) | Repeated measures | Biodex stability system - dynamic posturography tests | No-brace vs brace treatment Injured vs non-injured limb | Knee brace improved significantly the overall stability index (OSI) for **injured leg**. ^+veˆ^ No significant difference for the **non-injured le**g. ^-ve^ |
|  | Papadopoulos et al. (2007) | Repeated measures | Tekscan Mat | No-brace vs brace with 30kPa pressure vs brace with 60kPa pressure treatments. | Ankle brace treatments (30 & 60kPa pressure) resulted in a deterioration of the anteroposterior CoP excursion and excursion velocity, both with open and closed eyes. ^-ve^ |
|  | Sperlich et al. (2013) | Repeated measures | Moving platform (Posturomed) | No compression garment vs 20 mmhg compression garment vs 40 mmhg compression garment treatments. | No significant effect on single leg standing balance on the moving platform. ^-ve^ Compression garments in the range of 20-30mmhg may improve alpine skiing performance by allowing a deeper tuck position and lowering perceived exertion. ^+ve^ |
|  | Vuillerme & Pinsault (2007) | Repeated measures | Force platform (PF01, Equi+, Aix les Bains) | No tactile vs tactile (wide strips) treatment Non-fatigue vs fatigue conditions | Strips of athletic tape reduced the CoP surface area in both non-fatigue and fatigue conditions. ^+ve^ |
| Wearable garments | Wheat et al. (2014) | Repeated measures | Force platform (Kistler) | socks with no nodules vs nodules on the plantar surface vs nodules on the dorsal surface vs nodules at the side of the foot vs nodules for the entire surface | There was no statistically significant effect of adding texture to socks at any location on balance during single-legged standing in young, healthy adults. Results revealed a trend towards improved balance in the Sides sock condition. ^~^ |
|  | Woo et al. (2014) | Repeated measures | Force platform (Kistler) | Barefoot vs commercial socks vs compression socks | There were no significant main effects for the lightly-textured compression socks in postural control. ^-ve^ |
| Textured Materials | Aruin & Kaneka. (2013) | Repeated measures | SMART EquiTest System (NeuroCom International) GAITRite system | No insole vs right D-insole vs left D-insole | Significant immediate effect of the textured insole was seen in the outcome measures of static (weight bearing) and dynamic (weight symmetry index, strength symmetry) balance tests as well as in gait symmetry (single support and swing phases). ^+ve^ |
|  | Collings et al. (2015) | Repeated measures | Plantar pressure measurement system (Fscan, Teskcan)6 Telemetry unit (MT8) | No dowels insoles vs 2mm dowels insoles vs 4mm dowels insoles vs 6mm dowels insoles | The rate of CoP change was greater while wearing the lateral zoned insoles. This suggest that the site of stimulation of the plantar foot may increase postural instability during walking. ^~^ |
|  | Corbin et al. (2007) | Repeated measures | Accusway Plus Balance platform (Advanced Mechanical Technology) | Non-textured vs textured insoles | Postural control improved while subjects wore textured insoles during bilateral, eyes-closed stance. ^+ve^ There was no significant main effect for texture insoles in unilateral stance balancing task. ^-ve^ |
|  | Hatton et al. (2009) | Repeated measures | Force platform (Kistler) BIOPAC EMG system | Control insoles vs texture 1 (small pyramidal peaks) vs textured 2 (larger convex circular pattern | There was no significant effect of the two textured surfaces on postural balance. ^-ve^ |
|  | Hatton et al. (2011) | Repeated measures | Force platform (Kistler) BIOPAC EMG system | Control insoles (smooth) vs texture 1 (Evalite Pyramid EVA) vs textured 2 (nora Lunasoft Mini Non Slip) | Textured surfaces can improve ML sway in older people. A significant decrease in Medio-lateral range with eyes closed was observed when standing on texture 1. ^+ve^ |
|  | Hatton et al. (2012) | Repeated measures | Force platform (Kistler) GAITRite instrumentation (CIR system) | Control insoles (smooth) vs textured insoles | There was no significant difference of the textured insole on standing balance. ^-ve^ Textured insole leads to a significant reduction in gait velocity, step length and stride length. ^+ve^ |
|  | Ma et al. (2016) | Repeated measures | Balance Master1 system (NeuroCom International, USA).  GaitRite1 walkway | Single insoles vs no insole | There was no significant difference of the textured insole on the gait velocity and cadence.~  Asymmetry of stance and gait was observed immediately the implementation of the single textured insole. ~ |
|  | Maki et al. (1999) | Repeated measures | Horizontal translation of a computer-controlled, movable platform. | No tubing vs flexible polyethylene tubing | The facilitation (flexible polyethylene tubing) affected the responses of healthy young adults, as well as older subjects, high- lights the potent influence of these receptors. ~ Flexible polyethylene tubing appeared to improve control of stepping and postural reactions evoked by the continuous-perturbation platform. +ve Flexible polyethylene tubing decreased backward CoP excursion in feet-in-place response. ^+ve^ |
|  | Menz et al. (2006) | Mixed-Design | Optical displacement device (MEL Mikroelektronik, M5L/200) | No tactile stimulus vs tactile stimulus (Velcro) to the ankle vs tactile stimulus to the calf vs tactile stimulus to the knee | Passive tactile stimulus applied to the skin of the leg significantly reduces postural sway in healthy young subjects, older subjects without diabetic peripheral neuropathy and diabetic peripheral neuropathy patients. ^+ve^ |
|  | Palluel et al. (2008) | Mixed-design | Force platform (PF01, Equi+, Aix les Bains) | Non-spike insoles vs spike insoles | Spike insoles contributed to the improvement of unperturbed stance in the elderly with relatively intact plantar cutaneous sensation. ^+ve^ Standing or walking for 5 min lead to a significant improvement of balance in both elderly and young adults. For elderly, the effects were more pronounced in the standing than in the walking session. ^+ve^ |
| Textured Materials | Palluel et al. (2009) | Mixed-design | Force platform (PF01, Equi+, Aix les Bains) | Non-spike insoles vs spike insoles | Spike stimulation enhanced the somatosensation. The artificial sensory message elicited by the spikes improve postural control in both elderly and younger groups. ^+ve^ |
|  | Perry et al. (2008) | Mixed-design | Motion-analysis system (Optotrak 3020) | Conventional insoles vs facilitatory insoles (raised ridge) | The facilitatory insole improved lateral stability during gait. ^+ve^ Facilitatory-insole group appeared to reduce the fall rate. ^+ve^ |
|  | Qiu et al. (2012) | Mixed-design | Force platform (HUR Labs OY, Finland) | Barefoot vs hard textured insole surface (320 density ethhylenevinyl acetate) vs soft textured insole surface | The older group benefitted from the use of different insoles surfaces (hard & soft) on C90 area sway. ^+ve^ There was a significant and progressive decrease in path length from the barefoot to hard and to soft insole surface. ^+ve^ Both hard and soft insoles decrease the AP and ML sway for the older group when standing on foam surfaces. ^+ve^ |
|  | Qu (2015) | Repeated measures | Force platform (OR6-7, AMTI) | Cupped insoles vs textured insoles vs rigid insoles vs soft insoles | Static postural stability was not affected by the selected insoles. ^-ve^  Textured insoles did not improve static and dynamic balance. ^-ve^ |
|  | Stern & Gottschall (2012) | Repeated measures | 3D photogrammetric system (Motion Analysis Corporation) Wire amplifier system (Bortec Octopus AMT-8) | Regular insoles vs foam (Dr.Scholl's 2X Air Pillow insoles) vs textured vs barefoot vs Iced barefoot | Footwear conditions alter gait patterns. ^~^  There was not enough evidence to support that textured insoles improve dynamic balance. ^~^ |
|  | Wilson et al. (2008) | Mixed-design | Force platform (Kistler) GAITRite instrumentation (CIR system) | Control insoles vs Plain and smooth surface insoles vs dimpled surface insoles vs raised grid pattern insoles | No significant results were found. When shoe type is standardized there is no detrimental effect of postural ability over a 4-week period of intervention in the middle-age women using foot orthotic with differently textured surfaces. ^-ve^ |
| Stochastic Resonance | Amiridis et al. (2005) | Mixed-Design | Force platform (Kistler) | No electrostimulation vs electrostimulation (20-70mA) | CoP maximum displacement in anterio-posterior and mediolateral directions significantly decreased after electrostimulation training. ^+ve^ A reduction in anterioposterior sway. ^+ve^ |
|  | Collins et al. (2011) | Repeated measures | Force platform | No electrical stimulation and no sleeve (1) vs no electrical stimulation with sleeve vs 75% electrical stimulation with sleeve vs 150% electrical stimulation with sleeve vs no electrical stimulation and no sleeve (2) | No significant differences were found in the study measures between the testing conditions. ^-ve^  The SR electrical stimulation did not produce significant improvements in balance relative to the sleeve-alone condition. ^-ve^ |
|  | Dickstein et al. (2005) | Repeated measures | Force platform (AMTI) | Non-transcutaneous electrical nerve stimulation (TENS) vs Bilateral TENS vs TENS Left vs TENS Right | Application of TENS at threshold amplitudes to the posterior aspect of the calves reduces postural sway velocity (Decrease of 5%). ^+ve^ |
|  | Gravelle et al. (2002) | Repeated measures | Forceplate (Kistler 9286) | No electrical noise vs electrical noise( Gaussian white) | Overall improvement in balance performance in the electrical noise stimulation condition. ^+ve^ Three of the sway parameters (ML SD; AP Max; Path Length) decreased significantly in the electrical noise stimulation condition. ^+ve^ |
|  | Kimura & Kouzaki (2013) | Repeated measures | Force platform (EFP-S- 1.5kNSA13B) | No electrical stimulation vs electrical stimulation (white-noise-like, frequency 5-1000hz) | Postural sway reduced during quiet bipedal stance for electrical stimulation group. The average amplitude, the peak-to-peak amplitude, the SD of CoP(A/p) significantly decreased by 7.7%, 8.9% and 8.4% respectively. ^+ve^ |
|  | Magalhaes & Kohn (2011) | Repeated measures | Force platform (AMTI) | No electrical stimulation vs electrical stimulation (white-noise) to tibialis anterior (TA) vs electrical stimulation to triceps surae (TS) vs electrical stimulation to TA & TS | Electrical noise applies bilaterally over the TS muscles reduces postural oscillations during bipedal quiet stance. ^+ve^  There was a significant correlation between the reduction in force fluctuation and the decrease in postural sway due to the electrical noise stimulation. ^+ve^ |
| Stochastic Resonance | Magalhaes & Kohn (2014) | Repeated measures | Force platform (AMTI) | No electrical stimulation vs electrical stimulation (white-noise, 0.90ST) to tibialis anterior (TA) vs electrical stimulation to triceps surae (TS) vs electrical stimulation to TA & TS | Subsensory electrical noise applied to the anterior or posterior leg muscles significantly reduced postural sway measures when compared to control condition. ^+ve^ |
|  | Ross (2007) | Repeated measures | Force platform (Bertec) | No stochastic resonance (SR) stimulation vs SR stimulation (0.05mA or 0.01mA) | Optimal SR stimulation improved postural stability of functional ankle instability (FAI) group compared to the control condition. Significant reduction in centre-of-pressure velocities- resultant (COPV-R). ^+ve^ |
|  | Ross & Arnold (2012) | Repeated measures | Force platform (Bertec) | No stochastic resonance (SR) stimulation vs SR stimulation (0.05mA) | Stochastic resonance stimulation improved dynamic single leg balance by reducing anterioposterior Time-to-stabilisations (TTS) in FAI participants. ^+ve^ |
|  | Ross & Guskiewicz (2006) | Mixed-Design | Force platform (Bertec) | Coordination training vs SR stimulation (0.05mA) and coordination training. | SR stimulation might improve dynamic postural stability more quickly than coordination training for FAI participants. ^~^ |
|  | Ross et al. (2007) | Mixed-Design | Force platform (Bertec) | No stochastic resonance (SR) stimulation and coordination training vs coordination training vs SR stimulation (0.05mA) and coordination training. | SR stimulation used as an adjunct therapy to coordination training enhanced postural stability deficits associated with FAI. ^+ve^  Stochastic resonance and coordination training group had lower CoP velocity in AP and ML directions. ^+ve^ |
|  | Ross et al. (2013) | Mixed-Design | Accusway Plus Balance platform (Advanced Mechanical Technology) | 0% SR stimulation vs 25% SR stimulation vs 50% SR stimulation vs 75% SR stimulation vs 90% SR stimulation | Customized optimal intensity significantly improved double-leg standing balance in both FAI and healthy groups. ^+ve^ |

^+ve^ favor intervention; ^-ve^ favor control; ^~^ neutral
